# Identifying potential mechanisms between childhood trauma and the psychological response to the COVID‐19 pandemic in Germany: a longitudinal study

**DOI:** 10.1038/s41598-022-13205-1

**Published:** 2022-07-28

**Authors:** Stephanie V. Rek, Matthias A. Reinhard, Markus Bühner, Daniel Freeman, Kristina Adorjan, Peter Falkai, Frank Padberg

**Affiliations:** 1 Department of Psychiatry and Psychotherapy, University Hospital, Ludwig Maximilians University of Munich, Nussbaumstraße 7, Munich, Germany; 2 International Max Planck Research School for Translational Psychiatry (IMPRS-TP), Munich, Germany; 3 Department of Psychology, LMU Munich, Munich, Germany; 4 Department of Psychiatry, University of Oxford, Oxford, UK

**Keywords:** Psychology, Risk factors

## Abstract

Childhood maltreatment (CM) has been associated with adverse psychosocial outcomes during the pandemic, but the underlying mechanisms are unclear. In a prospective online study using baseline and 10-week follow-up data of 391 German participants, we applied multiple mediation analyses to test to what extent COVID-19 perceived stressors mediate the association between CM and later adverse psychosocial outcomes compared to established mediators of rumination and insecure attachment. We also explored the relative importance of different COVID-19 related stressors in predicting adverse psychological trajectories using elastic net regression. Results showed that CM was longitudinally associated with all adverse psychosocial outcome. COVID-19 perceived stressors, rumination, and insecure attachment mediated this relationship and full mediation was observed for the outcomes anxiety, stress and psychological well-being. COVID-19-related concerns about the future was most strongly and consistently associated with adverse psychosocial functioning. These findings provide preliminary evidence that COVID-19 perceived stressors, in particular concerns about the future, may be a key mechanism underlying the development of adverse psychosocial outcomes in individuals with a CM history. Thus, COVID-19 perceived stressors may require a higher priority for prevention and treatment efforts in vulnerable groups. Our results warrant replication in more representative cross-cultural samples.

## Introduction

It is obvious that the COVID-19 pandemic and its associated social restrictions placed an exceptional strain on individuals leading to a deterioration of mental health and well-being world-wide^1^. Specifically, the pandemic has resulted in unprecedented major stressors that can pose enormous psychological challenges including a virtual standstill of our public and private lives, anxieties about getting infected, the course of disease, and receiving appropriate medical care as well as but not limited to job uncertainties and financial difficulties. In a recent study, we could show that a greater impact of such COVID-19-specific stressors during the pandemic was associated with increased psychological difficulties in a German general population sample^2^. Moreover, representative cohort studies comparing changes in individuals before versus in the first few weeks of the initial lockdowns have suggested significant increases in mental health symptomatology^3,4^, also summarised in a recent meta-analysis^5^. In addition, longitudinal studies have identified heterogeneous trajectories of mental health symptomatology during the pandemic. Here, younger age, female sex, lower income levels, economic inactivity, and pre-existing mental health conditions have been associated with worse longitudinal psychological trajectories in terms of depression, anxiety, and loneliness (e.g.^6–11^). This demonstrates the importance of inter-individual differences in mental health trajectories and emphasises that identification of important risk factors and accompanying underlying mechanisms is key, which could allow for targeted care or prevention approaches.

One group of individuals that may be particularly vulnerable to the effects of the COVID-19 pandemic on mental health are those who experienced childhood maltreatment^12^. Childhood maltreatment (CM), which includes traumatic experiences of abuse and neglect, is arguably the most consistent transdiagnostic risk factor across psychiatric disorders and lower psychological well-being as shown in in multiple retrospective case–control and longitudinal studies (e.g.^13–19^). In the context of the COVID-19 pandemic, CM exposed individuals showed greater mental health difficulties in terms of symptoms of anxiety, depression, and posttraumatic stress disorder (PTSD), compared to non-exposed individuals in initial cross-sectional^20–23^ and longitudinal^24^ studies. However, not all CM exposed individuals develop mental health difficulties in adulthood and, so far, little is known about the exact pathways through which CM leads to an increased mental health risk^14^. In order to improve treatment or even prevent an adverse mental health cascade during the current pandemic, it is therefore crucial not only to identify vulnerable groups by environmental stratification based on CM criteria, but also to deepen our understanding of potential core mechanisms linking psychopathology to CM.

Stress sensitisation by early exposure to CM has been proposed as a key transdiagnostic mechanism leading to the evolvement of later psychopathology^25–28^. In the current pandemic, for instance, CM exposed individuals may be sensitised and particularly reactive to stress, which could lead to the perception that COVID-19-related stressors are particularly stressful. In turn, this could increase levels of adverse psychosocial outcomes. We are aware of only one longitudinal study amidst the current COVID-19 pandemic that showed that perceived stress mediated the association of early life adversity and depressive symptom severity in adolescents^29^. Yet, this study did not differentiate stressors specific versus unspecific to the COVID-19 pandemic. Further, it is unclear whether this mediation generalises to mental health conditions other than depression, and if it also occurs in adults. Finally, it is unclear to what extent perceived stress still plays a relevant mechanistic role when compared to established transdiagnostic mediators between CM and mental health such as rumination (e.g.^14,30–32^) and insecure attachment^33,34^. This can be tested using multiple mediation analyses adjusting for important confounding factors such as age, sex, income, educational attainment, and pre-existing mental health conditions.

This prospective study in individuals from the general population aims at investigating the relationship between CM and subsequent psychopathology and psychological well-being as well as the relative mediation via COVID-19 perceived stressors, rumination, and insecure attachment. Based on previous research and the theoretical considerations described above, we hypothesised (i) that CM is associated with more adverse psychosocial outcomes in terms of depression, anxiety, stress, loneliness, paranoia, and psychological well-being, and (ii) that these associations are mediated by COVID-19 perceived stressors, rumination, and insecure attachment. Understanding the factors linking higher rates of mental health difficulties during the current pandemic to CM can inform the development of targeted prevention and psychosocial treatment efforts. Since not all COVID-19 stressors may be equally important in predicting adverse psychosocial outcomes, we further explored their relative importance.

## Methods

### Participants and procedure

A longitudinal survey in German language was conducted in adults (18+ years old) with varying levels of CM who were recruited online via social media platforms and university mailing lists (see Supplementary Material for study advertisement text). The secure online survey software (LimeSurvey) was used for assessments, which was set up using a forced response format to prevent missing data and questionnaire block randomisation to circumvent potential carry over effects. As a reimbursement participants could be included in a prize draw by entering their email address at the end of the survey. Recruitment and initial assessment took place between April 2020 and May 2021 with follow-up assessment 10 weeks after the initial assessment. Participants completed a range of questionnaires (see Supplementary Table S1 for an overview of all the questionnaires), we only selected a subset of questionnaires, which we deemed relevant to answer our research questions.

All participants provided informed consent prior to participation, the study was conducted in accordance with the Declaration of Helsinki^35^ and approved by the Faculty of Medicine of the Ludwig-Maximilians-University Research Ethics Committee [Project Number: 20-118].

### Measures

#### Exposure (at baseline)

##### Childhood trauma questionnaire (CTQ)

CM was assessed by the CTQ self-report questionnaire that comprises five subscales: emotional, physical, and sexual abuse as well as emotional and physical neglect (Ref.^36^; German version^37^). Each subscale consists of five items, which are rated on a 5-point Likert scale ranging from 1 (not at all) to 5 (very much). Reversed items were recoded. The subscale scores were calculated to represent a total score. Scores on the total scale range from 25 to 125 with higher scores indicating more severe CM. In previous research good psychometric properties of the questionnaire have been reported^36^ and internal consistency of the total score at baseline was excellent in the present study (Cronbach’s α = 0.92).

#### Proposed mediators (at baseline)

##### COVID-19-specific stressor impact index

The COVID-19-specific stressor impact index of the COVID-19 Pandemic Mental Health Questionnaire (CoPaQ)^38^ was used to assess COVID-19 perceived stressors over the past 2 weeks. The subscale includes different COVID-19 stressors (e.g., quarantine/curfew, small accommodation/home-office, financial difficulties, childcare responsibilities, and physical health concerns), which are rated on a 5-point Likert scale ranging from 0 (not at all) to 4 (very much). The “not applicable” answer option was recoded as zero. Scores are summed to form a total score, which can range from 0 to 56 and higher scores indicate greater COVID-19 perceived stressors. Preliminary psychometric evaluation of this subscale was reported to be sound^39^. Internal consistency was not assessed for the COVID-19-specific stressor impact index since stressors can occur relatively independent from each other.

##### Perseverative thinking questionnaire (PTQ)

Rumination was assessed with the PTQ^40^, which consists of 15 items rated on a 5-point Likert scale ranging from 0 (Never) to 4 (Almost always). Items on content-independent negative ruminative thinking can be summed to a total score, which can range from 0 to 60. Higher scores indicate higher levels of ruminative thinking. Ehring et al.^40^ reported good psychometric properties of the scale and we observed excellent internal consistency (α = 0.96).

##### Relationship styles questionnaire (RSQ)

We used the RSQ (Ref.^41^; German version^42^) to measure insecure attachment styles. The scale is comprised of 30 items, which are rate on a 5-point Likert scale ranging from 1 (not at all) to 5 (very much). Reversed items were recoded. Attachment avoidance and attachment anxiety were defined as proposed by Roisman et al.^43^ (Model 3A). At baseline, internal consistency estimates of the attachment avoidance and attachment anxiety subscales were good (α_Avoidance_ = 0.80; α_Anxiety_ = 0.80).

#### Psychosocial outcome variables (at 10-week follow-up)

##### Depression, anxiety and stress scales (DASS-21)

We used the DASS-21 to measure levels of depression, anxiety, and stress during the preceding week (Ref.^44^; German version^45^). Items are rated on a 4-point Likert scale of 0 (did not apply to me at all) to 3 (applied to me very much or most of the time). Subscale scores can each range from 0 to 21, respectively, and higher scores indicate greater levels of psychopathology. Scores of each subscale were multiplied by two in order to convert scores to the full DASS-42 version^46^. In clinical and non-clinical samples good psychometric properties of the scales have been reported^47^. In our study, internal consistency estimates ranged from acceptable to excellent for each subscale (α_Depression_ = 0.93, α_Anxiety_ = 0.79, and α_Stress_ = 0.89).

##### Revised-Green et al. paranoid thoughts scale (R-GPTS)

Paranoia over the past fortnight was assessed with the total score of the 18-item R-GPTS (Ref.^48^; German version^52^). Items are rated on a 5-point Likert scale ranging from 0 (not at all) to 4 (totally). Scores can range from 0 to 72; higher scores indicate higher levels of paranoia. Excellent psychometric properties of the scale have been reported^48^. The German version was translated from the original English version following common guidelines for forward and backward translation^49^. The final version was approved by one of the authors of the original version (D.F.). At T2, internal consistency of the total score was excellent (α = 0.91).

##### UCLA loneliness scale (UCLA-LS)

Loneliness was assessed using the UCLA-LS (Ref.^50^; German version^51^), which includes 20 items rated on a 5-point Likert scale ranging from 1 (not at all) to 5 (totally). Items that were reversed were recoded. The average score was built to represent the mean, with higher scores indicating greater loneliness. Good psychometric properties of the scale have been reported in previous research^51^. In our sample, internal consistency was excellent at the second assessment timepoint (α = 0.94).

##### WHO (five) well-being index (WHO-5)

Psychological well-being was assessed with the WHO-5 (Ref.^52,53^; German version^49,54^). Five items are rated on a 6-point Likert scale ranging from 0 (at no time) to 5 (all of the time). Items were summed to represent the total score, which can range from 0 to 25, higher scores indicate greater well-being. Sound psychometric properties of the scale have been reported in previous research^55^. In our sample, internal consistency was excellent at follow-up (α = 0.90).

### Statistical analyses

All analyses were conducted in R version 4.0.0^56^ with packages *psych* (version 1.8.12^57^), *lavaan* (version 0.6-3.1295^58^)*,* and *glmnet* (version 4.1-1^59^).

First, bivariate Pearson’s correlation coefficients and Chi-square tests (χ^2^) were conducted to test associations between variables of interest.

Second, multiple mediation analyses using the maximum likelihood estimator were performed. CM was used as predictor variable and depression, anxiety, stress, loneliness, paranoia, and psychological well-being as outcome variables in separate mediation analyses. Mediator variables of COVID-19 perceived stressors, rumination, and attachment (anxious- and avoidant) were included simultaneously in the analyses and were allowed to correlate with each other. Standard errors were calculated using 10,000 bootstrap samples because some variables did not fully adhere to a normal distribution. We report bias-corrected 95% bootstrapped confidence intervals (CI) for the total (c), direct (cʹ), and indirect effects. Subdivision of the total indirect effect to specific indirect effects allowed comparisons of the standardised effect sizes of each mediator. Standardised ordinary least squares regression coefficients are reported for all paths. To account for potential influences of age, sex, income, educational attainment, and pre-existing mental health conditions (as diagnosed by a doctor or therapist, see Supplementary Material for details), variables were included as covariates in each multiple mediation model. As a post-hoc sensitivity analysis, we also added relationship status as an additional covariate to each multiple mediation model, which we defined as follows: being in a relationship (married, partnership) versus not being in a relationship (divorced, widowed, single). Of note, since multiple comparisons were performed we controlled for false positive rate by using the Benjamini–Hochberg procedure^60^.

Finally, to explore the relative importance of each of the different perceived COVID-19 stressors contributing to the total COVID-19 stressor index, we performed elastic net regression analyses^61^ to explore the relative predictive value of individual stressors for adverse psychosocial functioning. This technique is an extension of ordinary least squares regression that better accounts for collinearity between baseline predictors and simplifies the statistical model using regularisation. It includes the two hyperparameters α (tuning parameter of 0 to 1, which controls the type of shrinkage and, thus, the estimation method) and λ (penalty parameter of 0 to 1, which controls the amount of shrinkage with higher values leading to greater penalisation). The optimal hyperparameter combination was determined using grid search by selecting the model with smallest root mean squared error (RMSE) within tenfold cross-validation. Of note, regularisation with α = 1 equals Least Absolute Shrinkage and Selection Operator (LASSO) regression^62^ and α = 0 equals Ridge^63^ regression; α values between 0 to 1 reflect the relative balance between the two regression models. Elastic net regression models were controlled for age and sex.

## Results

### Sample characteristics

Six hundred sixty-eight participants completed the survey at baseline (T1) and 429 at the 10-week follow-up assessment (T2). To ensure high data quality, we excluded participants at baseline who answered more than 1 bogus item incorrectly (e.g., not checking “very much” for the item “Please, indicate ‘very much’”) (n = 58). In addition, participants with response times less than 25 min at baseline (n = 8) and less than 15 min at follow-up (n = 8) were excluded, which were deemed unlikely response times on the basis of personal experiences and response time descriptive statistics (baseline response time: median = 48 min, 1st quartile = 38 min, 3rd quartile = 61 min; follow-up: median = 23 min, 1st quartile = 29 min, 3rd quartile = 40 min). We also removed 30 participants who did not have a matching id variable between baseline and follow-up assessment, which resulted from a rare failure of the software. Taken together, this led to a final sample of 391 individuals (77.43% females) on which analyses are based (age: mean = 30.99, standard deviation[sd] = 11.52). The final sample consisted of 91.82% participants who indicated German nationality, 47.83% were single (see Table 1 for more demographic and clinical characteristics). Of note, participants only completing the baseline assessment did not differ significantly from the follow-up sample in terms of age, sex, nationality, employment status, marital status, and pre-existing mental health conditions (p > 0.05; see Supplementary Table S2).

**Table 1:** Baseline characteristics of analytic sample.

	Descriptive statistics
Sample size, *n*	391
Age, *mean (SD)*	30.99 (11.52)
Women sex, *n (%)*	303 (77.49)
**Employment status**^**a**^**, *n (%)***	
Full-time employed	88 (22.51)
Part-time employed	63 (16.11)
Self-employed	14 (3.58)
Student	185 (47.31)
Retired	7 (1.79)
Caregiver	0 (0)
Not employed	11 (2.81)
Other	23 (5.88)
**Freely disposable money per month, *n (%)***	
< 100 €	28 (7.16)
100–250 €	84 (21.48)
250–500 €	111 (28.39)
500–1000 €	85 (21.74)
> 1000 €	83 (21.23)
**Educational attainment, *n (%)***	
Primary school	0 (0)
Secondary school	39 (9.97)
A-levels	352 (90.03)
**Self-reported lifetime diagnoses, *n (%)***	
Number of diagnoses	
0	261 (66.75)
1	73 (18.67)
2	40 (10.23)
3	13 (3.32)
≥ 4	4 (1.02)
**Diagnostic categories**	
Depressive disorders	90 (23.02)
Bipolar disorders	3 (0.77)
Psychotic disorders	0 (0)
Anxiety disorders	45 (11.51)
Post-traumatic stress disorder	22 (5.63)
Obsessive–compulsive and related disorders	7 (1.79)
Eating disorders	18 (4.60)
Substance-related and addictive disorders	4 (1.02)
Attention-Deficit/hyperactivity disorder	9 (2.30)
Somatoform disorders	2 (0.51)
Personality disorders	9 (2.30)
Autism spectrum disorder	3 (0.77)
Dementia	0 (0)

^a^Employment status was assessed in forced choice format, so participants had to indicate the option they identified with most.

### Multiple mediation models

Descriptive statistics of proposed exposure, mediator, and outcome variables can be found in Table 2. As a prerequisite for mediation analyses, we observed bivariate correlations between all proposed exposure and mediator variables (path a), mediator and outcome variables (path b), and exposure and outcome variables (path c) as shown in Supplementary Tables S3 and S4. For example, COVID-19 perceived stressors were significantly associated with the predictor CM (r = 0.26, p < 0.01) and the criterion depression severity (r = 0.35, p < 0.01). Rumination was significantly related to the predictor (r = 0.27, p < 0.01) and criterion variable (r = 0.50, p < 0.01). Attachment anxiety was also significantly linked to CM (r = 0.25, p < 0.01) and depression severity (r = 0.32, p < 0.01), as was attachment avoidance (r_CM_ = 0.32; p < 0.01; r_Depression_ = 0.34, p < 0.01). Lastly, depressive symptom severity was significantly associated with CM severity (r = 0.35; p < 0.01).

**Table 2:** Descriptive statistics of exposure, proposed mediator, and psychosocial outcome variables.

	Mean (SD)	Range	IQR
**Exposure variable**			
T1 childhood maltreatment (CTQ)	37.55 (12.93)	25–100	29–43
**Proposed mediators**			
T1 COVID-19 perceived stressors (COPAQ)	13.63 (8.95)	0–47	7–20
T1 rumination (PTQ)	27.70 (14.00)	0–59	17–38
T1 anxious attachment	2.13 (0.91)	1–5	1.40–2.8
T1 avoidant attachment	2.64 (0.77)	1–4.50	2–3.13
**Criterion variables**			
T2 depression (DASS-21)	11.47 (10.75)	0–42	4–16
T2 anxiety (DASS-21)	5.86 (6.86)	0–36	0–8
T2 stress (DASS-21)	12.75 (9.77)	0–40	4–20
T2 loneliness (UCLA-LS)	2.19 (0.72)	1–4.45	1.55–2.70
T2 paranoia (R-GPTS)	8.33 (10.06)	0–57	1.5–11
T2 well-being (WHO-5)	12.21 (5.71)	0–25	7.50–17

*SD* standard deviation, *IQR* inter quartile range.

**Figure 1:**
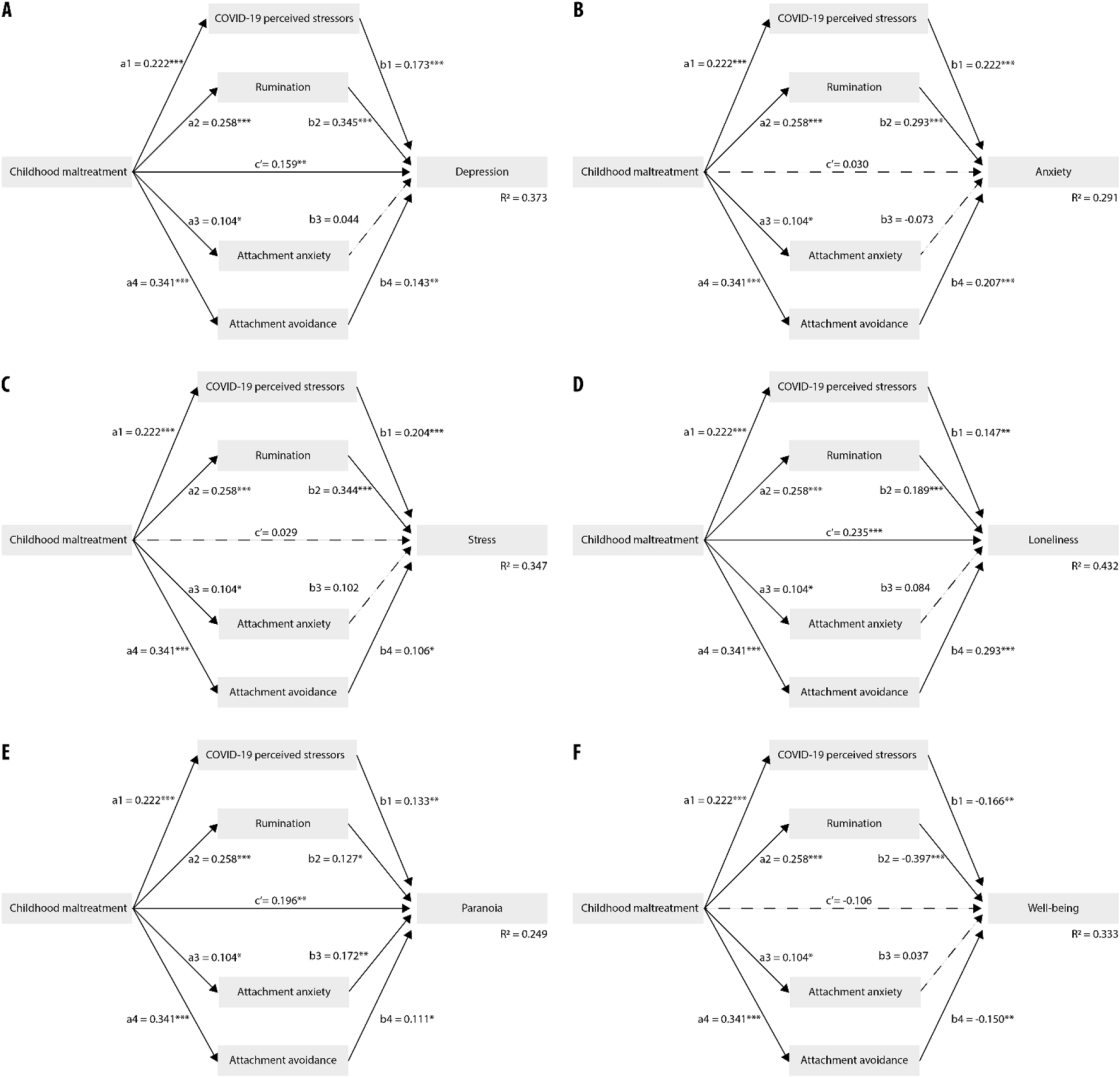
Multiple mediation models. The figure shows path diagrams for multiple mediation models for outcomes depression (**A**), anxiety (**B**), stress (**C**), loneliness (**D**), paranoia (**E**), and psychological well-being (**F**). Non-significant paths are visualised using dashed lines. Regression coefficients are standardised.

In separate multiple mediation models and as indicated by the total indirect effect, we observed evidence supporting overall mediation of effects of CM on the different outcome variables (i.e., depression, anxiety, stress, paranoia, loneliness, and psychological well-being) via COVID-19 perceived stressors, rumination, and attachment (anxious and avoidant) (see Table 3, Fig. 1). Specific indirect effects of the proposed mediators were significant for COVID-19 perceived stressors, rumination, and attachment avoidance across outcome variables. Attachment anxiety did not contribute additionally to the indirect effect in five out of six separate multiple mediation analyses; except paranoia. Full mediation between CM and anxiety as well as stress was observed as the direct effect (cʹ) was no longer significant after inclusion of the proposed mediators. Partial mediation was observed for the relationship between CM and depression, paranoia, loneliness, and psychological well-being since the direct effect remained significant. This suggests that our proposed set of mediators did not fully explain this relationship. Of note, all results from multiple mediation analyses were adjusted for the potential confounders age, sex, income, educational attainment, and pre-existing mental health conditions and the mediators were allowed to correlate with each other (Supplementary Table S5 shows additional adjustment of relationship status, which did not alter the pattern of results substantially). When applying alpha correction for multiple testing, results remained largely unchanged but the specific indirect effect of attachment avoidance was no longer significant for the outcomes stress and paranoia. Moreover, full mediation was observed for psychological well-being (see Table 3).

**Table 3:** Multiple mediation models with standardised bootstrap intervals.

DV	Std. point estimate	SE	p	p_corrected_	CI Lower	CI Upper	R^2^
**Depression**							
Total (c)	0.340	0.050	< 0.001	< 0.001	0.185	0.383	0.373
Total indirect	0.181	0.027	< 0.001	< 0.001	0.100	0.206	
Specific indirect							
COVID-19 perceived stressors	0.038	0.012	0.009	0.014	0.012	0.060	
Rumination	0.089	0.018	< 0.001	< 0.001	0.041	0.113	
Attachment anxiety	0.005	0.005	0.474	0.511	− 0.006	0.016	
Attachment avoidance	0.049	0.015	0.008	0.013	0.013	0.073	
Direct (cʹ)	0.159	0.050	0.007	0.012	0.037	0.228	
**Anxiety**							
Total (c)	0.218	0.037	0.002	0.004	0.039	0.183	0.291
Total indirect	0.188	0.016	< 0.001	< 0.001	0.068	0.130	
Specific indirect							
COVID-19 perceived stressors	0.049	0.009	0.005	0.009	0.011	0.047	
Rumination	0.075	0.011	< 0.001	0.001	0.019	0.063	
Attachment anxiety	− 0.008	0.005	0.392	0.433	− 0.015	0.003	
Attachment avoidance	0.070	0.011	< 0.001	0.001	0.018	0.059	
Direct (cʹ)	0.030	0.036	0.658	0.674	− 0.057	0.084	
**Stress**							
Total (c)	0.210	0.044	< 0.001	< 0.001	0.070	0.243	0.347
Total indirect	0.181	0.025	< 0.001	< 0.001	0.088	0.186	
Specific indirect							
COVID-19 perceived stressors	0.045	0.012	0.005	0.009	0.014	0.061	
Rumination	0.089	0.016	< 0.001	< 0.001	0.038	0.101	
Attachment anxiety	0.011	0.006	0.172	0.201	− 0.001	0.021	
Attachment avoidance	0.036	0.013	0.044	0.057	0.001	0.055	
Direct (cʹ)	0.029	0.044	0.615	0.646	− 0.065	0.107	
**Loneliness**							
Total (c)	0.425	0.003	< 0.001	< 0.001	0.019	0.032	0.432
Total indirect	0.190	0.002	< 0.001	< 0.001	0.008	0.015	
Specific indirect							
COVID-19 perceived stressors	0.033	0.001	0.010	0.015	0.001	0.004	
Rumination	0.049	0.001	0.003	0.006	0.001	0.005	
Attachment anxiety	0.009	< 0.001	0.204	0.231	< 0.001	0.002	
Attachment avoidance	0.100	0.001	< 0.001	< 0.001	0.004	0.009	
Direct (cʹ)	0.235	0.003	< 0.001	< 0.001	0.008	0.02	
**Paranoia**							
Total (c)	0.314	0.054	< 0.001	< 0.001	0.136	0.347	0.249
Total indirect	0.118	0.021	< 0.001	< 0.001	0.051	0.134	
Specific indirect							
COVID-19 perceived stressors	0.030	0.010	0.023	0.032	0.006	0.046	
Rumination	0.033	0.013	0.054	0.067	0.001	0.053	
Attachment anxiety	0.018	0.008	0.085	0.102	0.001	0.032	
Attachment avoidance	0.038	0.015	0.047	0.060	0.001	0.059	
Direct (cʹ)	0.196	0.053	0.004	0.008	0.046	0.255	
**Psychological well-being**							
Total (c)	− 0.292	0.024	< 0.001	< 0.001	− 0.176	− 0.082	0.333
Total indirect	− 0.186	0.014	< 0.001	< 0.001	− 0.112	− 0.057	
Specific indirect							
COVID-19 perceived stressors	− 0.037	0.006	0.010	0.014	− 0.031	− 0.006	
Rumination	− 0.102	0.011	< 0.001	< 0.001	− 0.068	− 0.027	
Attachment anxiety	0.004	0.003	0.555	0.583	− 0.004	0.008	
Attachment avoidance	− 0.051	0.008	0.005	0.009	− 0.040	− 0.008	
Direct (cʹ)	− 0.106	0.024	0.047	0.060	− 0.092	0.001	

Depicted are total, total indirect, specific indirect, and direct effects of the different multiple mediation models.

*DV* dependent variable, *CI* confidence interval (bootstrapped), *Std.* standardised, *P*_*corrected*_ false discovery rate corrected p value.

### Elastic net regression

**Figure 2:**
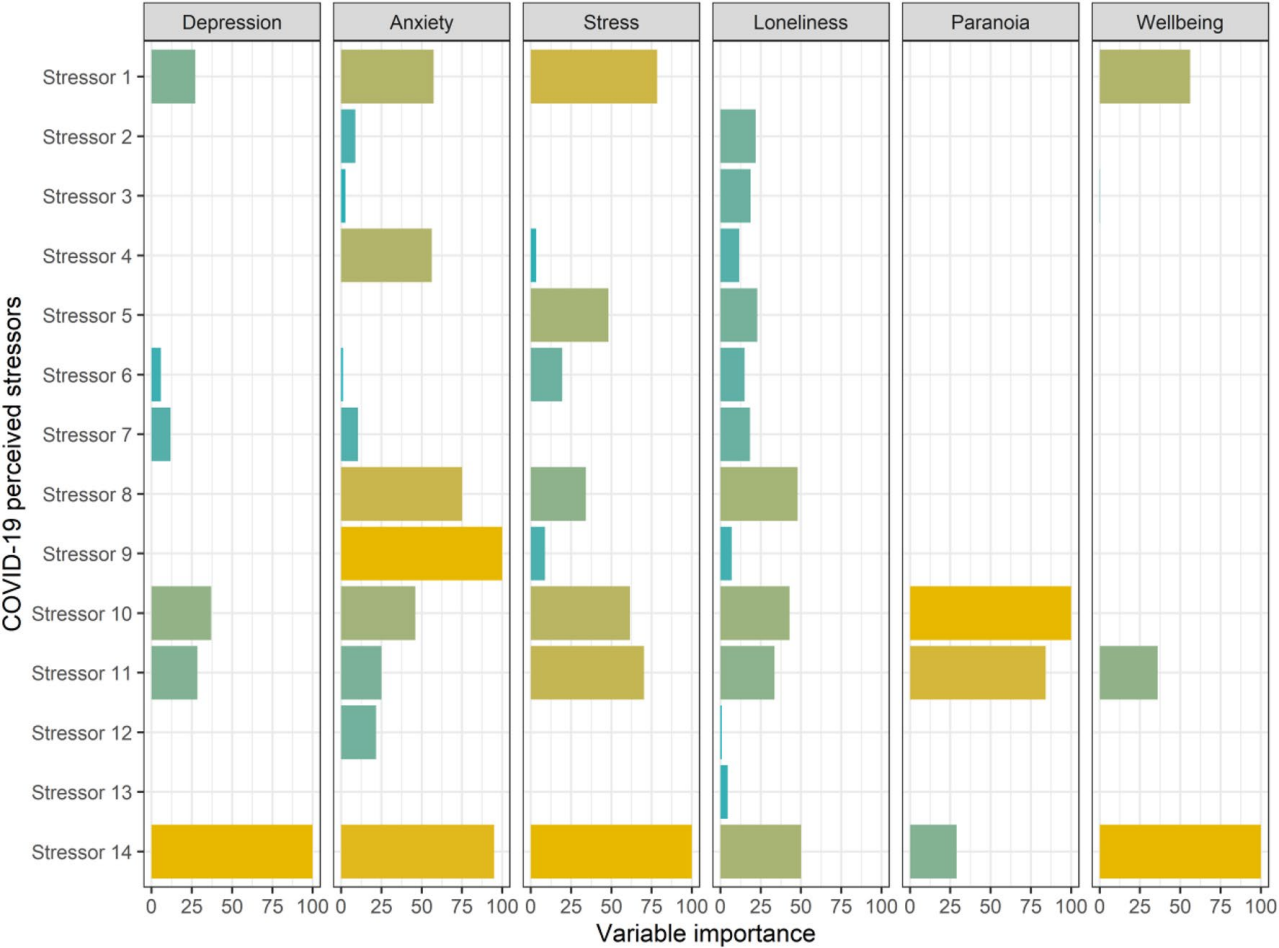
Relative importance of COVID-19 related stressors in predicting psychosocial outcomes. Relative importance was extracted from elastic net regression models with optimal hyperparameters selected during cross-validation (see Supplementary Table S6). Analyses were adjusted for age and sex. Stressor 1 = the current pandemic, Stressor 2 = living in a small accommodation, Stressor 3 = being in quarantine, Stressor 4 = childcare, Stressor 5 = taking over school lessons, Stressor 6 = the curfew, Stressor 7 = being in home office, Stressor 8 = customer service, Stressor 9 = worries about my health, Stressor 10 = worries of not being able to get medical care, Stressor 11 = increased conflicts with people close to me, Stressor 12 = financial worries, Stressor 13 = uncertainties regarding my job, training place, studies or school, Stressor 14 = fears of what the future will bring, or that I won't be able to cope with everything.

We explored the relative variable importance of different COVID-19 stressors in predicting psychosocial outcomes using elastic net regression and results from the selected models are depicted in Fig. 2. Of note, the selected elastic net regression model for outcome loneliness was ridge regression (α = 0), so none of the predictor variables were set exactly to zero, and LASSO (α = 1) for outcomes stress, paranoia, and wellbeing. The hyperparameter α was in between 0 and 1 for outcomes depression and anxiety reflecting the optimal balance between ridge and LASSO regression selected for these outcomes during cross-validation. Of note, analyses were adjusted for age and sex. See Supplementary Table S6 for hyperparameters of selected models.

A few COVID-19 stressors were rather consistently associated with adverse outcomes. Among them were “the current pandemic” (stressor 1), “worries of not being able to get medical care” (stressor 10), “increased conflicts with people close to me” (stressor 11), and “fears of what the future will bring, or that I won’t be able to cope with everything” (stressor 14). In contrast, stressors such as “living in a small accommodation” (stressor 2), “being in home office” (stressor 7), and “uncertainties regarding my job, training place, studies or school” (stressor 13) were consistently unpredictive of adverse psychosocial outcomes. For some outcomes, the impact of COVID-19 perceived stressors was predominately associated with a few stressors that explained most of the variance. For example, levels of psychological well-being were only predicted by three COVID-19 stressors, namely “the current pandemic” (stressor 1), “increased conflicts with people close to me” (stressor 11), and “fears of what the future will bring, or that I won't be able to cope with everything” (stressor 14). For other psychosocial outcomes such as anxiety and loneliness, variable importance was rather equally distributed across stressors.

## Discussion

This prospective study scrutinised the pathway between CM and multiple key psychosocial outcomes (depression, anxiety, stress, paranoia, loneliness, and psychological well-being) during the COVID-19 pandemic regarding potential mediators: (1) COVID-19 perceived stressors, (2) rumination and (3) insecure attachment while adjusting for important confounders of age, sex, income, educational attainment, and pre-existing mental health conditions. Our findings showed that COVID-19 perceived stressors may be a robust mechanism mediating the effect between CM and adverse psychosocial outcomes during the current pandemic. We also observed that rumination and insecure attachment (particularly attachment avoidance) acted as important additional mediators of the relationship between CM and psychosocial outcomes. The relative contribution of each potential mediator suggested that rumination was most strongly associated with adverse psychosocial outcomes. Of note, full mediation was observed for anxiety, stress and psychological well-being but not depression, paranoia, and loneliness. Taken together, our study adds to the accumulating research that highlights the importance of considering early adverse experiences when evaluating the psychological response to the current pandemic^21–23,28,62^. In addition, our findings suggest two important modifiable therapeutic targets, i.e. rumination and COVID-19 perceived stressors, the latter being specific to the pandemic for prevention and treatment efforts.

Our findings correspond well to the stress sensitisation hypothesis^27^, which argues that early adverse experiences lead to stress sensitisation making individuals more reactive to later stressors and, thus, increasing the susceptibility for adult psychopathology. Indisputable, the current pandemic and associated countermeasures pose a heavy strain on many individuals with anxieties about the disease, temporary closures of educational institutes, enforced social isolation, job losses, and financial difficulties to name only a few of the many stressors associated with the pandemic. Our findings show that the subjective perception of such COVID-19 related stressors matters in the relationship between CM and later adverse psychosocial outcomes during the pandemic. Moreover, we show that the impact of some stressors may be more relevant than others. Here, our explorative analyses highlight that perceived stress related to the pandemic itself, interpersonal conflicts, concerns about medical care resources, and worries about the future were among the stressors that mattered most for almost all adverse psychosocial outcomes. In sum, these results advance previous research on the relationship between CM and later adverse psychosocial outcomes by quantifying the relevance of perceived stressors specific to the pandemic^29^.

For the outcomes of depression, loneliness, and paranoia, we observed only partial mediation suggesting that other mechanisms may be relevant to explain the relationship between early CM experiences and later adverse psychosocial outcomes. In our study, we focused on relatively established subjective psychological mediators of perceived stressors (specific to the COVID-19 pandemic), rumination, and insecure attachment. Yet, there may be other potentially relevant psychological but also biological mediators of the relationship that we did not assess and which may help to identify additional factors that are amenable to treatment. Psychologically, for instance, recent research has highlighted the importance of perceived lack of social support during the current pandemic^64^ and its transdiagnostic importance has also been discussed before the pandemic^14^. Biologically, proinflammatory processes have been implicated in depressive symptomology specifically^65,66^ and are proposed to explain the link between CM and later depression^67–69^. As such, our study may have benefitted from inclusion of these factors and future longitudinal research should test their relative relevance in mediating the relationship between CM and later psychopathology in order to identify specific factors that can be therapeutically targeted.

### Strengths and limitations

Strengths of the present study include (i) the prospective study design, (ii) assessment of perceived stressors specifically related to the COVID-19 pandemic, (iii) simultaneous integration of established mediators of insecure attachment and rumination, (iv) sophisticated analytical techniques, and (v) inclusion of a broad array of key psychosocial outcomes. Yet, the study also has some important limitations. First, assessments are based exclusively on self-report questionnaires, so future research would benefit from inclusion of observer-based ratings, for example, by conducting structured interviews to assess attachment using the gold standard Adult Attachment Interview^70^ or the Adult Attachment Projective^71^. Second, although we report prospective data, only two timepoints of a 10-week time window were included and CM was assessed retrospectively. As several studies have shown a discrepancy between prospective and retrospective measures of CM^72,73^, one has to be aware that CM scores always represent a subjective recall of adverse events during childhood which does not mean that this recall is invalid or less relevant. However, to minimise a potential recall bias, for example, due to current mood, we adjusted for self-reported life time mental health diagnoses. Yet, there may be other important confounders, for which we did not control. We also did not assess the specific forms, duration, or frequency of CM, which may be important moderators but beyond the scope of the present study. Third, assessments of COVID-19-specific stressors and mental health outcomes were made between April 2020 and May 2021, when the COVID-19 pandemic and related countermeasures varied in Germany (ranging from lockdowns to easing of restrictions to distribution of vaccines). This changing external context and the associated psychological response could confound analyses and complicates pinpointing the exact impact of specific stressors over time. Finally, we report data of an online survey unrepresentative of the German population, in which female sex and relatively young participants were overrepresented. Therefore, replication in more representative or cross-national samples would allow for greater generalisability of findings.

## Conclusion

The current prospective study investigated the impact of CM on later adverse psychosocial outcomes during the COVID-19 pandemic. After adjusting for key confounding variables, we showed that subjective perception of COVID-19 stressors matters in the psychological response to the pandemic in addition to more established mediators of rumination and insecure attachment. Our findings underscore the importance of stress sensitisation in childhood trauma-exposed individuals that is also present in the current pandemic. Importantly, identified mediators of COVID-19 perceived stressors, rumination, and insecure attachment fully explain the relationship between CM and anxiety, stress or psychological well-being and are amenable to psychological interventions. Thus, researchers and clinicians who encounter patients with a history of CM during the pandemic are advised to assess these key psychological mediators for more targeted prevention and treatment efforts in order to prevent deterioration of mental health.

## Supplementary Information

Supplementary Information.

## Data Availability

The datasets used and/or analysed during the current study are available from the corresponding author on reasonable request**.**
