# Supplementary material for: Identifying potential mechanisms between childhood trauma and the psychological response to the COVID‐19 pandemic in Germany: a longitudinal study
Source: Sci Rep. 2022 Jul 28;12:12964. doi: 10.1038/s41598-022-13205-1 (PMC9333057; doi:10.1038/s41598-022-13205-1)
Supplement: Supplementary file 1 — Supplementary Information. [file 41598_2022_13205_MOESM1_ESM.docx]

**Supplementary Material**

**Identifying Potential Mechanisms between Childhood Trauma and the Psychological Response to the COVID‐19 Pandemic in Germany: A Longitudinal Study**

Stephanie V. Rek^1,2^, Matthias A. Reinhard^1^, Markus Bühner^3^, Daniel Freeman^4^, Kristina Adorjan^1^, Peter Falkai^1^, & Frank Padberg^1^

^1^Department of Psychiatry and Psychotherapy, LMU University Hospital Munich, Munich, Germany

^2^International Max Planck Research School for Translational Psychiatry (IMPRS-TP), Munich, Germany

^3^Department of Psychology, LMU Munich, Munich, Germany

^4^Department of Psychiatry, University of Oxford, Oxford, UK

**Contents**

[Supplementary Table 1. Overview of all the questionnaires participants completed. 3](#_Toc101953241)

[Supplementary Methods 4](#_Toc101953242)

[Advertisement text 4](#_Toc101953243)

[Assessment of pre-existing mental health diagnosis 4](#_Toc101953244)

[Supplementary Table 2. Baseline characteristics stratified by follow-up participation 5](#_Toc101953245)

[Supplementary Table 3. Bivariate correlations between exposure, proposed mediator, and outcome variables 6](#_Toc101953246)

[Supplementary Table 4. Bivariate correlations between exposure and proposed mediator variables 7](#_Toc101953247)

[Supplementary Table 5. Multiple mediation models with additional adjustment of relationship status with standardised bootstrap intervals 8](#_Toc101953248)

[Supplementary Table 6. Elastic net hyperparameters selected during cross-validation 9](#_Toc101953249)

# Supplementary Table 1. Overview of all the questionnaires participants completed

| **Socio-demographic questions** |
| --- |
| **Depression, Angst und Stress Skalen (DASS-21)**  *Lovibond, S.H. & Lovibond, P.F. (1995). Manual for the Depression Anxiety & Stress Scales. (2nd Ed.)Sydney: Psychology Foundation.* |
| **Child Trauma Questionnaire (CTQ)**  *Bernstein, D. P., Stein, J. A., Newcomb, M. D., Walker, E., Pogge, D., Ahluvalia, T. et al. (2003). Development and validation of a brief screening version of the Childhood Trauma Questionnaire. Child Abuse Negl., 27(2), 169-190.* |
| **Inventory of Interpersonal Problems-32 (IIP-32)**  *Horowitz, L. M., Rosenberg, S. E., Baer, B. A., Ureno, G., & Villasenor, V S. (1988). Inventory of interpersonal problems: Psychometric properties and clinical applications. Journal of Consulting and Clinical Psychology, 56, 885-892.* |
| [**Relationship Scales Questionnaire (RSQ)**](#_Toc524612628)  *Griffin, D. W., & Bartholomew, K. (1994). Models of the self and other: Fundamental dimensions underlying measures of adult attachment. Journal of Personality and Social Psychology, 67(3), 430.* |
| **Fragebogen zu belastenden Sozialerfahrungen in der Peergroup (FBS)**  *Sansen, L., Iffland, B., Catani, C., & Neuner, F. (2013). Entwicklung und Evaluation des Fragebogens zu belastenden Sozialerfahrungen in der Peergroup (FBS). Zeitschrift für Klinische Psychologie und Psychotherapie.* |
| **Loneliness Scale (UCLA)**  *Russell, D., Peplau, L. A., & Ferguson, M. L. (1978). Developing a measure of loneliness. Journal of personality assessment,42(3), 290-294.* |
| **Revised-Green et al Paranoid Thoughts Scale (R-GPTS)**  *Freeman, D., Loe, B. S., Kingdon, D., Startup, H., Molodynski, A., Rosebrock, L., Brown, P., Sheaves, B., Waite, F., Bird J. C. (accepted). The Revised Green et al Paranoid Thoughts Scale (R-GPTS): Psychometric Properties, Severity Ranges, and Clinical Cut-offs. Psychological Medicine.* |
| **Brief Chore Schema Scales (BCSS)**  *Fowler, D., Freeman, D., Smith, B. E. N., Kuipers, E., Bebbington, P., Bashforth, H., ... & Garety, P. (2006). The Brief Core Schema Scales (BCSS): psychometric properties and associations with paranoia and grandiosity in non-clinical and psychosis samples. Psychological Medicine, 36(6), 749-759.* |
| **Community Assessment of Psychic Experiences-P15 (CAPE-P15)**  *Stefanis NC, Hanssen M, Smirnis NK, Avramopoulos DA, Evdokimidis IK, Stefanis CN, Verdoux H, Van Os J (2002). Evidence that three dimensions of psychosis have a distribution in the general population.* Psychological Medicine*,* ***32:*** *347–358.* |
| **Cambridge Depersonalisation Scale (CDS)**  *Sierra, M., & Berrios, G. E. (2000). The Cambridge Depersonalisation Scale: A new instrument for the measurement of depersonalisation. Psychiatry research, 93(2), 153-164.* |
| **Perseverative Thinking Questionnaire (PTQ)**  *Ehring, T., Zetsche, U., Weidacker, K., Wahl, K., Schönfeld, S., & Ehlers, A. (2011). The Perseverative Thinking Questionnaire (PTQ): Validation of a content-independent measure of repetitive negative thinking. Journal of behavior therapy and experimental psychiatry, 42(2), 225-232.* |
| **Social Network Index (SNI)**  *Cohen, S., Doyle, W. J., Skoner, D. P., Rabin, B. S., and Gwaltney, J. M., Jr. (1997). Social ties and susceptibility to the common cold. Journal of the American Medical Association, 277, 1940-1944. Journal of the American Medical Association, 277, 1940-1944.* |
| **Brief Resilience Scale (BRS)**  *Smith, B. W., Dalen, J., Wiggins, K., Tooley, E., Christopher, P., & Bernard, J. (2008). The brief resilience scale: assessing the ability to bounce back. International journal of behavioral medicine, 15(3), 194-200.* |
| **COVID-19 Pandemic Mental Health Questionnaire (CoPaQ)**  *Rek, S. V., Bühner, M., Reinhard, M. A., Freeman, D., Keeser, D., Adorjan, K., ... & Padberg, F. (2021). The COVID-19 Pandemic Mental Health Questionnaire (CoPaQ): psychometric evaluation and compliance with countermeasures in psychiatric inpatients and non-clinical individuals. BMC psychiatry, 21(1), 1-15.* |

# Supplementary Methods

## Advertisement text

The study was advertised as the following:

“My Network, Others and Resilience (NEAR) - Survey during Corona Times:

Dear Participant,

Thank you for your interest in our survey. We invite you to take part in an online survey on relationships and resilience, which is being conducted by the Ludwig Maximilian University of Munich. The aim of the study is to investigate, by means of various questionnaires, what influence (possibly stressful) relationship experiences in the past and/or present have on our psychological well-being. For this we would like to ask for your help. If you are interested, please click on the following link to the survey:”

## Assessment of pre-existing mental health diagnosis

Using “Yes” or “No” as answer options we asked participants the following question:

“Have you ever been diagnosed by a doctor or therapist with one or more of the following?

a) Depression

b) Mania/Bipolar disorder

c) Psychotic disorders (including schizophrenia)

d) Anxiety disorder

e) Posttraumatic stress disorder

f) Eating disorder

g) Compulsive disorders (OCD)

h) Substance abuse or Addiction disorder

i) Attention disorder (ADD or ADHD)

j) Somatoform disorder

k) Personality disorder

l) Autism Spectrum Disorder (including Asperger’s Syndrome)”

The variable was subsequently dummy coded to represent either having received (1) versus not having received a mental health diagnosis (0) by a doctor or therapist.

# Supplementary Table 2. Baseline characteristics stratified by follow-up participation

|  | Non-Completers | Completers | p value |
| --- | --- | --- | --- |
| n | 211 | 391 |  |
| Age, *mean (SD)* | 30.47 (11.65) | 30.99 (11.52) | 0.600 |
| Women sex, *n (%)* | 170 (80.6) | 303 (77.5) |  |
| Nationality, *n (%)* | 187 (88.6) | 359 (91.8) | 0.105 |
| Marital status, *n (%)* |  |  |  |
| Married | 38 (18.0) | 65 (16.6) | 0.976 |
| Partnership | 72 (34.1) | 141 (36.1) |  |
| Single | 93 (44.1) | 172 (44.0) |  |
| Divorced | 6 (2.8) | 9 (2.3) |  |
| Widowed | 2 (0.9) | 4 (1.0) |  |
| Employment status, *n (%)* | | |  |
| Full-time employed | 50 (23.7) | 88 (22.5) | 0.082 |
| Part-time employed | 33 (15.6) | 63 (16.1) |  |
| Self-employed | 6 (2.8) | 14 (3.6) |  |
| Student | 85 (40.3) | 185 (47.3) |  |
| Retired | 4 (1.9) | 7 (1.8) |  |
| Caregiver | 4 (1.9) | 0 (0.0) |  |
| Not employed | 9 (4.3) | 11 (2.8) |  |
| Other | 20 (9.5) | 23 (5.9) |  |
| Self-reported lifetime diagnoses, *n (%)* |  |  |  |
| Diagnostic categories | | |  |
| Depressive Disorders | 49 (23.2) | 90 (23.0) | 1 |
| Bipolar Disorders | 3 (1.4) | 3 (0.8) | 0.733 |
| Psychotic Disorders | 7 (3.3) | 0 (0.0) | 0.001 |
| Anxiety Disorders | 29 (13.7) | 45 (11.5) | 0.505 |
| Post-Traumatic Stress Disorder | 14 (6.6) | 22 (5.6) | 0.751 |
| Eating Disorders | 15 (7.1) | 18 (4.6) | 0.271 |
| Obsessive-Compulsive and Related Disorders | 2 (0.9) | 7 (1.8) | 0.645 |
| Substance-Related and Addictive Disorders | 7 (3.3) | 4 (1.0) | 0.092 |
| Attention-Deficit/Hyperactivity Disorder | 9 (4.3) | 9 (2.3) | 0.272 |
| Somatoform Disorders | 2 (0.9) | 2 (0.5) | 0.918 |
| Autism Spectrum Disorder | 2 (0.9) | 3 (0.8) | 1 |
| Personality Disorders | 9 (4.3) | 9 (2.3) | 0.272 |
| Dementia | 0 (0) | 0 (0) | 1 |

# Supplementary Table 3. Bivariate correlations between exposure, proposed mediator, and outcome variables

| Exposure and mediator variables at baseline | Outcome at 10-week follow-up | | | | | |
| --- | --- | --- | --- | --- | --- | --- |
|  | Depression | Anxiety | Stress | Paranoia | Loneliness | Well-being |
|  |  |  |  |  |  |  |
| Childhood Maltreatment | .35** | .24** | .25** | .32** | .42** | -.30** |
|  | [.26, .43] | [.14, .33] | [.15, .34] | [.23, .40] | [.34, .50] | [-.38, -.20] |
|  |  |  |  |  |  |  |
| COVID-19 Perceived Stress | .35** | .35** | .37** | .27** | .30** | -.32** |
|  | [.26, .43] | [.26, .44] | [.29, .46] | [.18, .36] | [.21, .39] | [-.41, -.23] |
|  |  |  |  |  |  |  |
| Rumination | .50** | .45** | .53** | .34** | .40** | -.50** |
|  | [.43, .57] | [.37, .52] | [.45, .59] | [.25, .43] | [.31, .48] | [-.57, -.42] |
|  |  |  |  |  |  |  |
| Avoidant Attachment | .34** | .33** | .29** | .29** | .46** | -.32** |
|  | [.25, .42] | [.24, .42] | [.20, .38] | [.19, .38] | [.38, .54] | [-.40, -.22] |
|  |  |  |  |  |  |  |
| Anxious Attachment | .32** | .24** | .37** | .33** | .31** | -.25** |
|  | [.22, .40] | [.14, .33] | [.28, .45] | [.24, .42] | [.21, .39] | [-.34, -.15] |
|  |  |  |  |  |  |  |

*Note.* Values in square brackets indicate the 95% confidence interval for each correlation. The confidence interval is a plausible range of population correlations that could have caused the sample correlation (Cumming, 2014). * indicates *p* < .05. ** indicates *p* < .01.

# Supplementary Table 4. Bivariate correlations between exposure and proposed mediator variables

| Variable | 1 | 2 | 3 | 4 |
| --- | --- | --- | --- | --- |
| 1. Childhood Maltreatment |  |  |  |  |
| 2. COVID-19 Perceived Stress | .26** |  |  |  |
|  | [.19, .34] |  |  |  |
| 3. Rumination | .27** | .36** |  |  |
|  | [.20, .34] | [.29, .43] |  |  |
| 4. Attachment Avoidance | .32** | .11** | .31** |  |
|  | [.24, .39] | [.03, .19] | [.23, .38] |  |
| 5. Attachment Anxiety | .25** | .33** | .47** | .31** |
|  | [.18, .33] | [.26, .40] | [.41, .53] | [.24, .38] |
|  |  |  |  |  |

*Note.* *M* and *SD* are used to represent mean and standard deviation, respectively. Values in square brackets indicate the 95% confidence interval for each correlation. The confidence interval is a plausible range of population correlations that could have caused the sample correlation (Cumming, 2014). * indicates *p* < .05. ** indicates *p* < .01.

| DV | Std. point estimate | SE | p | p_corrected_ | CI Lower | CI  Upper | R^2^ |
| --- | --- | --- | --- | --- | --- | --- | --- |
| **Depression** |  |  |  |  |  |  |  |
| Total (c) | 0.339 | 0.050 | <0.001 | <0.001 | 0.185 | 0.382 | 0.373 |
| Total indirect | 0.179 | 0.027 | <0.001 | <0.001 | 0.099 | 0.206 |  |
| Specific indirect |  |  |  |  |  |  |  |
| COVID-19 perceived stressors | 0.039 | 0.012 | 0.009 | 0.013 | 0.012 | 0.060 |  |
| Rumination | 0.090 | 0.018 | <0.001 | <0.001 | 0.042 | 0.113 |  |
| Attachment anxiety | 0.004 | 0.005 | 0.492 | 0.530 | -0.006 | 0.016 |  |
| Attachment avoidance | 0.047 | 0.016 | 0.015 | 0.021 | 0.010 | 0.073 |  |
| Direct (c’) | 0.16 | 0.050 | 0.007 | 0.012 | 0.037 | 0.229 |  |
| **Anxiety** |  |  |  |  |  |  |  |
| Total (c) | 0.218 | 0.037 | 0.002 | 0.004 | 0.039 | 0.182 | 0.291 |
| Total indirect | 0.188 | 0.016 | <0.001 | <0.001 | 0.068 | 0.130 |  |
| Specific indirect |  |  |  |  |  |  |  |
| COVID-19 perceived stressors | 0.049 | 0.009 | 0.005 | 0.009 | 0.011 | 0.047 |  |
| Rumination | 0.076 | 0.011 | <0.001 | 0.001 | 0.019 | 0.063 |  |
| Attachment anxiety | -0.008 | 0.005 | 0.395 | 0.437 | -0.016 | 0.003 |  |
| Attachment avoidance | 0.070 | 0.011 | <0.001 | 0.001 | 0.018 | 0.058 |  |
| Direct (c’) | 0.031 | 0.036 | 0.658 | 0.658 | -0.057 | 0.085 |  |
| **Stress** |  |  |  |  |  |  |  |
| Total (c) | 0.214 | 0.044 | <0.001 | <0.001 | 0.074 | 0.245 | 0.357 |
| Total indirect | 0.186 | 0.025 | <0.001 | <0.001 | 0.091 | 0.191 |  |
| Specific indirect |  |  |  |  |  |  |  |
| COVID-19 perceived stressors | 0.045 | 0.012 | 0.005 | 0.009 | 0.014 | 0.061 |  |
| Rumination | 0.086 | 0.016 | <0.001 | <0.001 | 0.036 | 0.098 |  |
| Attachment anxiety | 0.011 | 0.006 | 0.155 | 0.181 | -0.001 | 0.022 |  |
| Attachment avoidance | 0.044 | 0.014 | 0.015 | 0.021 | 0.007 | 0.061 |  |
| Direct (c’) | 0.028 | 0.044 | 0.633 | 0.649 | -0.066 | 0.106 |  |
| **Loneliness** |  |  |  |  |  |  |  |
| Total (c) | 0.424 | 0.003 | <0.001 | <0.001 | 0.019 | 0.032 | 0.431 |
| Total indirect | 0.188 | 0.002 | <0.001 | <0.001 | 0.008 | 0.015 |  |
| Specific indirect |  |  |  |  |  |  |  |
| COVID-19 perceived stressors | 0.033 | 0.001 | 0.009 | 0.014 | 0.001 | 0.004 |  |
| Rumination | 0.050 | 0.001 | 0.002 | 0.005 | 0.001 | 0.005 |  |
| Attachment anxiety | 0.008 | <0.001 | 0.219 | 0.248 | <0.001 | 0.001 |  |
| Attachment avoidance | 0.096 | 0.001 | <0.001 | <0.001 | 0.003 | 0.009 |  |
| Direct (c’) | 0.236 | 0.003 | <0.001 | <0.001 | 0.008 | 0.020 |  |
| **Paranoia** |  |  |  |  |  |  |  |
| Total (c) | 0.316 | 0.054 | <0.001 | <0.001 | 0.137 | 0.349 | 0.257 |
| Total indirect | 0.122 | 0.021 | <0.001 | <0.001 | 0.053 | 0.138 |  |
| Specific indirect |  |  |  |  |  |  |  |
| COVID-19 perceived stressors | 0.029 | 0.01 | 0.024 | 0.031 | 0.006 | 0.045 |  |
| Rumination | 0.031 | 0.013 | 0.070 | 0.087 | -0.001 | 0.051 |  |
| Attachment anxiety | 0.018 | 0.008 | 0.082 | 0.098 | 0.001 | 0.033 |  |
| Attachment avoidance | 0.044 | 0.015 | 0.020 | 0.028 | 0.006 | 0.064 |  |
| Direct (c’) | 0.194 | 0.053 | 0.004 | 0.008 | 0.045 | 0.255 |  |
| **Psychological well-being** |  |  |  |  |  |  |  |
| Total (c) | -0.292 | 0.024 | <0.001 | <0.001 | -0.175 | -0.082 | 0.333 |
| Total indirect | -0.185 | 0.014 | <0.001 | <0.001 | -0.111 | -0.056 |  |
| Specific indirect |  |  |  |  |  |  |  |
| COVID-19 perceived stressors | -0.037 | 0.006 | 0.008 | 0.013 | -0.03 | -0.006 |  |
| Rumination | -0.103 | 0.010 | <0.001 | <0.001 | -0.068 | -0.027 |  |
| Attachment anxiety | 0.004 | 0.003 | 0.543 | 0.570 | -0.004 | 0.008 |  |
| Attachment avoidance | -0.049 | 0.008 | 0.007 | 0.012 | -0.039 | -0.007 |  |
| Direct (c’) | -0.106 | 0.023 | 0.043 | 0.055 | -0.091 | <0.001 |  |

# Supplementary Table 5. Multiple mediation models with additional adjustment of relationship status with standardised bootstrap intervals

# Supplementary Table 6. Elastic net hyperparameters selected during cross-validation

| Outcome | α | λ |
| --- | --- | --- |
| Depression | 0.8 | 0.8 |
| Anxiety | 0.2 | 0.8 |
| Stress | 1.0 | 0.6 |
| Loneliness | 0.0 | 0.2 |
| Paranoia | 1.0 | 0.6 |
| Wellbeing | 1.0 | 0.4 |

*Note*. α = tuning parameter of 0 to 1. λ = penalty parameter of 0 to 1.
